# Thermal Induced Interface Mechanical Response Analysis of SMT Lead-Free Solder Joint and Its Adaptive Optimization

**DOI:** 10.3390/mi13060908

**Published:** 2022-06-08

**Authors:** Shaoyi Liu, Yuefei Yan, Yijiang Zhou, Baoqing Han, Benben Wang, Daxing Zhang, Song Xue, Zhihai Wang, Kunpeng Yu, Yu Shi, Congsi Wang

**Affiliations:** 1 Key Laboratory of Electronic Equipment Structure Design, Ministry of Education, Xidian University, Xi’an 710071, China; syliu_z@stu.xidian.edu.cn (S.L.); yjzhou3@stu.xidian.edu.cn (Y.Z.); hanbaoqing@xidian.edu.cn (B.H.); benbenwang@stu.xidian.edu.cn (B.W.); sxue@xidian.edu.cn (S.X.); 2 Guangzhou Institute of Technology, Xidian University, Guangzhou 510555, China; zhangdx@xidian.edu.cn; 3 CETC No. 38 Research Institute, Hefei 230088, China; ericwang@ustc.edu.cn (Z.W.); yukunp@hotmail.com (K.Y.); 4 Department of Mechanical Engineering, University of Chester, Thornton Science Park, Chester CH1 4BJ, UK; y.shi@chester.ac.uk

**Keywords:** SMT, cohesive zone model, contact pressure, Kriging, surrogate model, IEGO, adaptive optimization

## Abstract

Surface mount technology (SMT) plays an important role in integrated circuits, but due to thermal stress alternation caused by temperature cycling, it tends to have thermo-mechanical reliability problems. At the same time, considering the environmental and health problems of lead (Pb)-based solders, the electronics industry has turned to lead-free solders, such as ternary alloy Sn-3Ag-0.5Cu (SAC305). As lead-free solders exhibit visco-plastic mechanical properties significantly affected by temperature, their thermo-mechanical reliability has received considerable attention. In this study, the interface delamination of an SMT solder joint using a SAC305 alloy under temperature cycling has been analyzed by the nonlinear finite element method. The results indicate that the highest contact pressure at the four corners of the termination/solder horizontal interface means that delamination is most likely to occur, followed by the y-direction side region of the solder/land interface and the top arc region of the termination/solder vertical interface. It should be noted that in order to keep the shape of the solder joint in the finite element model consistent with the actual situation after the reflow process, a minimum energy-based morphology evolution method has been incorporated into the established finite element model. Eventually, an Improved Efficient Global Optimization (IEGO) method was used to optimize the geometry of the SMT solder joint in order to reduce the contact pressure at critical points and critical regions. The optimization result shows that the contact pressure at the critical points and at the critical regions decreases significantly, which also means that the probability of thermal-induced delamination decreases.

## 1. Introduction

**Figure 1:**
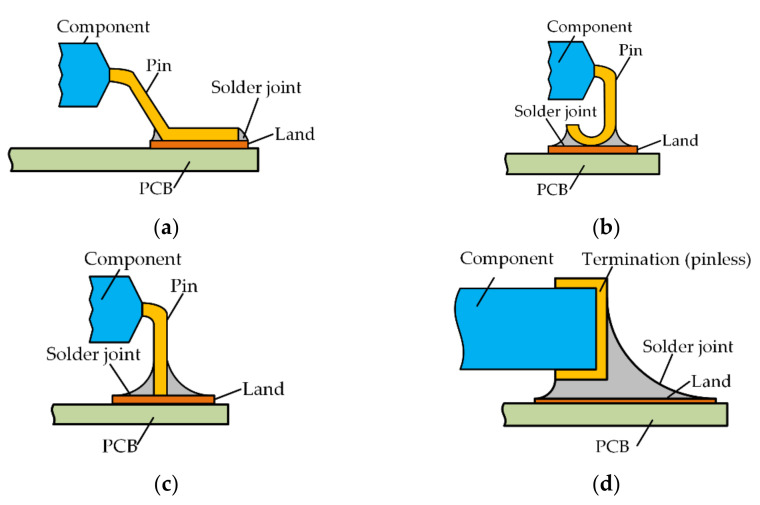
Pin forms of common SMT packages. (**a**) The gull-wing pin; (**b**) the I pin; (**c**) the J pin; (**d**) the pin-less form (using metal termination).

Electronic packaging technology not only defines the electrical signal and power path from the chip to the system but also plays the role of mechanical protection for IC components, which improves the environmental adaptability level of the circuit and guarantees continuous high performance. Surface Mount Technology (SMT) is the main form of board-level packaging. Compared with through-hole technology, the packaging size of SMT is greatly reduced, the operation is convenient, the reliability is high, and the installation wiring of the printed circuit board (PCB) is more convenient. It may contain dozens or even thousands of SMT solder joints on a complete board-level package circuit. The shape of SMT solder joints is affected by the pin shape of electronic components. There are many forms of pins, such as gull-wing pin, J pin, I pin and pin-less, as shown in Figure 1 [1]. Today, the most common type of solder joints are the pin-less solder joints.

However, electronic products are often in a complex service environment, such as high temperature, low temperature, high humidity, dust, vibration, salt mist and many other adverse environmental effects. When any of the solder joints in the circuit fails, it may cause the circuit to break down, causing the system to crash or the unstable connection of components to fall off. The main failure modes of solder joints are fatigue, creep, brittle fracture and plastic deformation, etc., but all of them are presented on the surface or inside of the solder joints in the form of interface delamination or crack. At present, crack failure analysis of solder joints is mainly carried out by the finite element method or experiments. Xiaoyan Li and Fenghui Li have studied the growth behavior of intermetallic compounds (IMC) in SAC305 solder joints and Cu lands during reflow and isothermal aging. During reflow, the grain size of Cu6Sn5 coarsens rapidly [2]. During isothermal aging, the growth rate of IMC increases with the increase in aging temperature. During thermal cycling, the high-temperature isothermal time has a significant effect on the growth rate of IMC. Yilong Chen and Jianyuan Jia et al. have studied experimentally the location of cracks at the interface of BGA solder joints caused by tensile stress [3]. They found that cracks not only occur at the interface between the solder and the land but also on the solder and the land itself. Zhen Liu et al. studied the crack behavior of chip on chip (COC) stacked interconnection microbump joint under drop impact load [4]. It was found that the crack leading to microbump failure first began at the interface between the IMC layer and solder. The effects of solder thickness and crack length on crack propagation were discussed. However, due to the miniaturization of the SMT component and time-consuming inspection, it is difficult to use an experimental method. With the development of the computer, numerical simulation using the finite element analysis (FEA) method is a more appropriate means of pre-analysis than testing. P. Rajaguru and H. Lu et al. explored the effect of the geometric parameters of the soldering layer on the ability of solder joints to withstand thermal cycling through the FEA method [5]. They found that the wetting angle and the thickness of the solder layer had positive effects on the solder’s ability to withstand thermal cycling, and the angle of inclination had negative effects on the solder layer’s ability to withstand thermal cycling. Luchun Yan et al. studied the cracking and failure of solder joints due to thermal mismatch using the FEA method [6]. The results show that the coefficients of thermal expansion mismatch between the solder and copper layer not only affected the maximum stress at the chip–solder interface but also led to the peak stress recovery. Z. Huang and P. Kumar et al. proposed a method to study the fracture behavior of solder joints with interface cracks under plane strain [7]. Using the linear expansion method of Crack Tip Opening Displacement (CTOD), the relationship between geometric factors and crack length was determined, and a function equation to calculate the critical crack length of the fracture test was obtained. Wenchao Tian et al. proposed a wafer-level, high-density microbump bonding technology and optimized the thickness, structure and layout of bonding materials by comparing and selecting good bonding processes [8,9,10]. Finally, they realized low-resistant electrical performance connections and obtained higher reliability, which is very useful for improving the reliability of 3D-system packaging. Their other study analyzed the stress–strain behavior of power semiconductor devices under temperature cycling, and what is of great value is that this study also optimized the solder overflow in the packaging process. Another exciting work is the study of the accurate characterization of the rough contact surface of microswitch by sine model and the analysis of its microscale effect.

Although the finite element software can simulate the stress and strain fields of SMT solder joints more accurately and conveniently, the FEA should simulate the actual situation as much as possible. From the existing research, there are the following main problems in the FEA of interface delamination or crack of SMT solder joints:

- The geometric model of SMT solder joints is greatly simplified. The reliability of SMT solder joints depends heavily on their geometry. SMT solder joints are often small in size. A tiny change in the solder joint morphology can lead to different simulation results.
- Most finite element analysis is assumed to be a plane stress or plane strain problem. The reliability analysis of SMT solder joints is inherently a three-dimensional problem. However, most current studies simplify the problem as a two-dimensional problem, which makes the analysis of the solder joint unreliable.
- An effective geometric shape optimization process is missing in the solder joint morphology design stage. Some existing studies use the Taguchi method to explore the optimal combination of geometric parameters [11,12,13]. However, the combination of optimization parameters is not necessarily globally optimal. Some other studies have introduced the numerical optimization algorithm, which has global optimization capability but is not suitable for high-cost problems, such as FEA, because of the large number of iterations [14,15].

**Figure 2:**
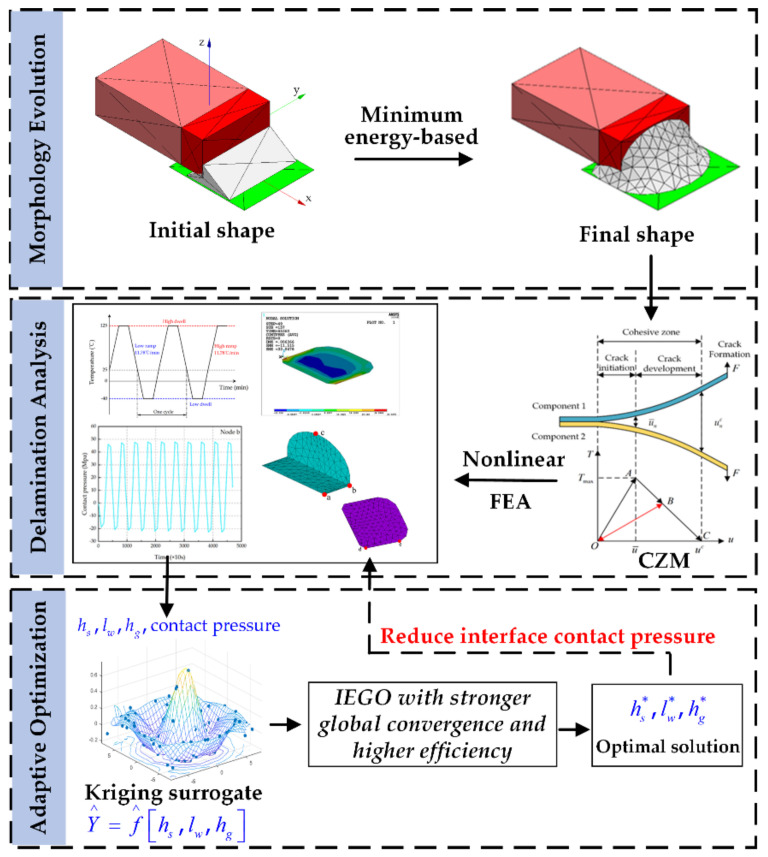
The framework of this paper.

To solve these problems, this paper has established the SMT solder joint model based on the minimum energy, which considers the constraints of external force, gravity and liquid tension when the solder alloy melts into a liquid state, and causes the model to possess the actual shape of the solder joint after the reflow process. It makes up for the imprecise solution caused by the large gap between the traditional simplified model and the actual solder joint shape. At the same time, a three-dimensional finite element (FE) model is established by using the Cohesion Zone Model (CZM), and CZM is used to solve the contact pressure between solder joint and termination, solder joint and land, so as to characterize the delamination trend of the solder joint interface. Finally, an Improved Efficient Global Optimization (IEGO) algorithm is presented, which is an adaptive global optimization algorithm based on the Kriging surrogate model. However, it has the advantage of invoking less expensive calculation times than traditional numerical optimization algorithms. Meanwhile, the improved infill criterion and convergence criterion make the algorithm more resistant to local optimals and converge faster. The framework of this paper is shown in Figure 2.

## 2. Numerical Model

### 2.1. CZM-Based Interfacial Delamination in Bi-Material Analysis Method

In traditional fracture mechanics theory, for linear elastic materials, the critical strength factor and critical energy release rate are taken as the basis for crack initiation. For nonlinear elastomers, the path-independent J-integral is used as the basis for crack initiation [16]. There are two types of problems with crack propagation:

1. An unknown crack propagation path requires the crack initiation force and crack propagation angle to be calculated by the crack propagation criterion.
2. For a known crack propagation path, only relevant criteria are needed to determine the initiation force.

The CZM is the main method used to study the second type of problem [17]. It avoids calculating the fracture parameters at the crack tip and does not require rigorous mesh at the crack tip. Therefore, the CZM is suitable for the study of solder joint interface delamination.

The interaction between microscopic particles within a material is the essence of cohesion [18]. The cohesion region represents the area along the front of the crack tip where the crack initiation is imminent. The crack in the region is not a real crack either. It is a virtual numerical crack, which is used to describe the stress field between two virtual crack interfaces.

Due to the different properties of materials, the tension–displacement relationship of cohesion elements varies, resulting in a variety of cohesive zone laws, which mainly belong to exponential, trapezoid and bilinear cohesive zone models. Bilinear CZM is used in this paper because of its stability and practicability [19].

**Figure 3:**
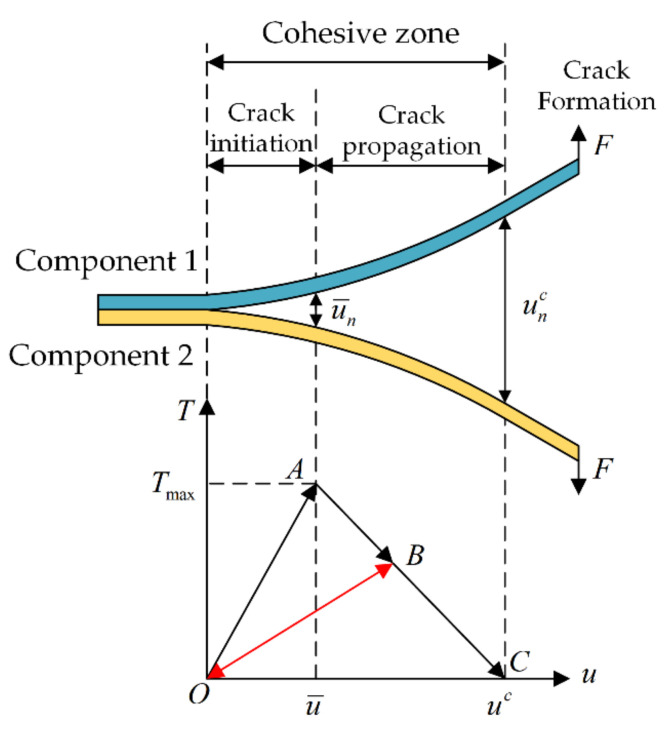
Cohesion zone and constitutive relationship.

Figure 3 shows the relationship between the contact stress and contact gap. The curve in the figure consists of a linear elastic load section OA and a subsequent linear softening section AC. Component 1 and component 2 are bonded together originally. In the curve OA section, the interface is subjected to external loads, and the internal contact stress T in the cohesion zone increases linearly with the increase in the contact gap u. When the internal contact stress reaches the maximum $T_{\max}$, the gap increases to $\bar{u}$, the material at the junction of the components is damaged, and the crack initiation begins. In the curve AC segment, the gap displacement increases continuously under the continuous action of external loads, but the internal contact stress T decreases, making it difficult for the material to resist the damage of external loads, leading to crack during the propagation stage. After point C, when the internal stress T decreases to 0, the gap increases to $u_{}^{c}$, the interior of the material has been completely destroyed, and the crack has been fully formed, at which point there will be no contact stress even if further separation occurs.

The area enclosed by the OAC curve is equal to the rate of energy release from the interface delamination, also known as the critical rate of energy release. When the curve changes from OA to OB, the quantity of delamination at the interface can be accumulated. The maximum subsequent tension can continue to delaminate the interface by only reaching the vertical coordinate of point B, without reaching the maximum tension at point A. The subsequent tension–displacement curve will be enclosed by a straight OB with a smaller slope and a straight BC.

The governing equation for the bilinear CZM is as follows:

(1) $$T_{n}=\left\{\begin{array}{cc} \frac{\sigma_{\max}}{{\bar{u}}_{n}}u_{n} & \left(u_{n}\leq {\bar{u}}_{n}\right)\\ \sigma_{\max}\frac{u_{n}^{c}-u_{n}}{u_{n}^{c}-{\bar{u}}_{n}}u_{n} & \left(u_{n}>{\bar{u}}_{n}\right) \end{array}\right.$$

(2) $$T_{t}=\left\{\begin{array}{cc} \frac{\tau_{\max}}{{\bar{u}}_{t}}u_{t} & \left(u_{t}\leq {\bar{u}}_{t}\right)\\ \tau_{\max}\frac{u_{t}^{c}-u_{t}}{u_{t}^{c}-{\bar{u}}_{t}}u_{t} & \left(u_{t}>{\bar{u}}_{t}\right) \end{array}\right.$$

where $T_{n}$ and $T_{t}$ are normal and tangential contact stress, $\sigma_{\max}$ and $\tau_{\max}$ are normal and tangential maximum contact stress, ${\bar{u}}_{n}$ and ${\bar{u}}_{t}$ are the contact gap under maximum normal and tangential contact stress, ${\bar{u}}_{n}^{c}$ and ${\bar{u}}_{t}^{c}$ are the maximum gap when the interface is delaminate under normal and tangential forces.

The normal and tangential critical energy release rates in the CZM are $G_{n}^{c}$ and $G_{t}^{c}$, which are defined as follows:

(3) $$G_{n}^{c}=\frac{\sigma_{\max}{\bar{u}}_{n}^{c}}{2}$$

(4) $$G_{t}^{c}=\frac{\tau_{\max}{\bar{u}}_{t}^{c}}{2}$$

### 2.2. Minimum Energy-Based SMT Solder Joint Geometry Modeling

The geometry of the solder is constrained by external forces, self-gravity, and liquid tension when it is in a melting liquid state. In order to keep the shape stable during the reflow process, the liquid solder will continuously evolve to the lowest energy state until the total energy is lowest; that is, when the energy of the solder system is lowest, the liquid solder will reach the static balance state. Compared with other mathematical analysis models, the minimum energy method can be used for more diverse, complex, and accurate analysis of solder shaping.

**Figure 4:**
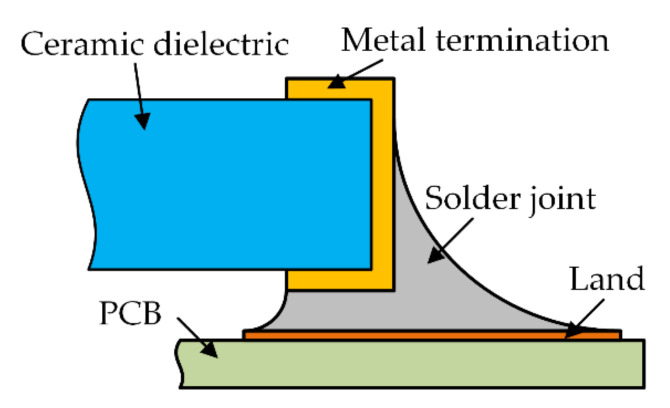
SMT solder joint structure sketch.

**Figure 5:**
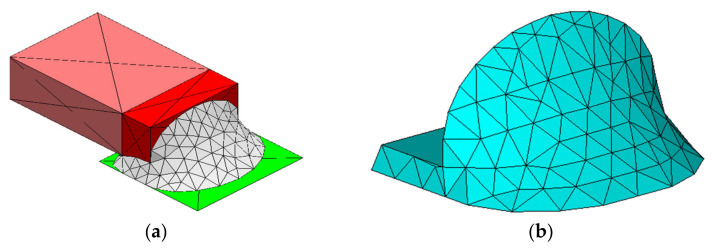
SMT solder joint geometry model based on minimum energy. (**a**) Solder joint geometry modeling evolved in surface evolver; (**b**) reconstructed in ANSYS.

This paper uses Surface Evolver FE simulation software (Version 2.70, the software can be downloaded from http://facstaff.susqu.edu/brakke/evolver/evolver.html, accessed on 16 May 2022) developed by Professor Kenneth. A. Brakke to solve the SMT solder joint shape [20]. This software is based on the principle of minimum energy and is often used to simulate the properties of a liquid static interface. The purpose of this paper is to propose a method that can guide the simulation analysis and adaptive optimization of contact pressure for any SMT device, but the ceramic surface mount capacitor 0805 is still the research object. There is metal wrap-around termination on the left and right sides of the ceramic dielectric. Below is PCB and metal land attached to them. Solder joints are bonded between metal pins and lands (half of the structure is modeled and analyzed due to the symmetrical structure of the surface mount capacitor). The structure sketch is shown in Figure 4. Details of energy and geometric constraints required for shape evolution in the surface evolver are given in Appendix A. The final shape of the SMT solder joint generated by evolution based on the principle of minimum energy is shown in Figure 5a, and the node coordinates and surface unit information of the three-dimensional model can be output through a .def format file. The ANSYS Parametric Design Language (APDL) command is used to reconstruct the model for finite element analysis. As shown in Figure 5a,b.

### 2.3. FEA Modeling Process

Capacitor, land, PCB and solder joints generated based on the principle of minimum energy are built into a complete geometric model of the SMT component, and then the material parameters of each part are set, meshed, and a complete FE model is built. For multilayer ceramic capacitors, some ideal simplification of the materials and structure of capacitor components is made: (a) Since more than 90% of the capacitor body is ceramic dielectric, the capacitor body is simplified as a solid filled with ceramic dielectric, and the internal electrodes and barrier layers are ignored; (b) merge the external electrodes, barrier layers and terminations. Set the land to be a flat copper pad, 1.3 mm in length, 1.1 mm in width and 0.05 mm in thickness. Materials other than solder joints are set to linear elastic behavior.

In the FEA of temperature cycling (TC), the FE model will be subjected to a thermal cycling condition of −45–125 °C, whereas the melting point of SAC305 solder is 217 °C, so the high dwell temperature exceeds 50% of the melting point of the solder alloy, resulting in high-temperature deformation of SAC305 solder, that is, elastic, plastic and creep deformation [21]. Therefore, Anand’s visco-plastic material model is used to accurately simulate the mechanical behavior of the SAC305 alloy under TC load in this paper.

Anand’s visco-plastic material model can be expressed by the following:

(5) $$\frac{d\varepsilon_{p}}{dt}=A\left(m\sqrt{\sin h\left(\zeta \sigma /s\right)}\right)\exp\left(\frac{Q}{kT}\right)$$

(6) $$\frac{ds}{dt}=\left\{h_{0}{\left|1-\frac{s}{s^{*}}\right|}^{a}\cdot sign\left(1-\frac{s}{s^{*}}\right)\right\}\cdot \overset{*}{\varepsilon_{p}}$$

(7) $$s^{*}=\hat{s}{\left[\frac{d\varepsilon /dt}{A}\exp\left(\frac{Q}{kT}\right)\right]}^{n}$$

Equation (5) defines the steady-state plastic flow equation of the solder joint. Equation (6) is the internal deformation impedance equation. Equation (7) is the calculation of the saturation value of the distortion impedance, where $d\varepsilon_{p}/dt$ is the equivalent plastic strain rate, A is the pre-exponential factor, m is the strain rate sensitive index, ζ is the stress multiplier, Q/k is the activation energy, $h_{0}$ is the hardening constant, a is the strain rate sensitive index, $s^{*}$ is the distortion impedance saturation value, $\hat{s}$ is the distortion impedance saturation value coefficient, n is the saturation value strain sensitive index. The parameters of Anand’s visco-plastic model for SAC305 are shown in Table 1. The remaining material parameters and other constants associated with the FE model are shown in Table 2.

**Table 1:** Anand model parameters for SAC305 [22].

Parameter Symbol	Description	Value
$s_{0}$ (MPa)	Initial value of deformation resistance	2.15
Q/k (K^−^^1^)	Activation energy	9970
A (s^−^^1^)	Pre-exponential factor	17.994
ζ (dimensionless)	Stress multiplier	0.35
m (dimensionless)	Strain rate sensitivity of stress	0.153
$h_{0}$ (MPa)	Hardening/softening constant	1525.98
$\hat{s}$ (MPa)	Coefficient for saturation value of deformation resistance	2.536
n (dimensionless)	Strain rate sensitivity of the saturation value	0.028
a (dimensionless)	Strain rate sensitivity of the hardening/softening	1.69

**Table 2:** Relevant material parameters in FEA [23,24,25,26].

Materials	$\rho \;({g/\mathrm{cm}}^{3})$	E (GPa)	ν	$\alpha \;(\times 10^{-6}/K)$
BaTiO3	6.02	76.5	0.32	8
SAC305	7.38	Table 3	0.36	Table 4
Cu	8.92	117.0	0.34	16.6
Epoxy	2.02	19.7	0.31	8.8 (x, y) 20 (z)

**Table 3:** Elastic modulus of SAC305 (temperature dependent).

Temperature (°C)	−65	−55	0	25	65	105	130
E (GPa)	59.0	56.7	43.8	37.4	28.6	20.5	13.5

**Table 4:** Coefficient of thermal expansion of SAC305 (temperature dependent).

Temperature (°C)	−55	−35	−15	5	22	50	75	100	125
$\alpha \;(\times 10^{-6}/K)$	21.1	21.6	22	22.2	22.4	23.1	23.7	24.3	24.9

**Figure 6:**
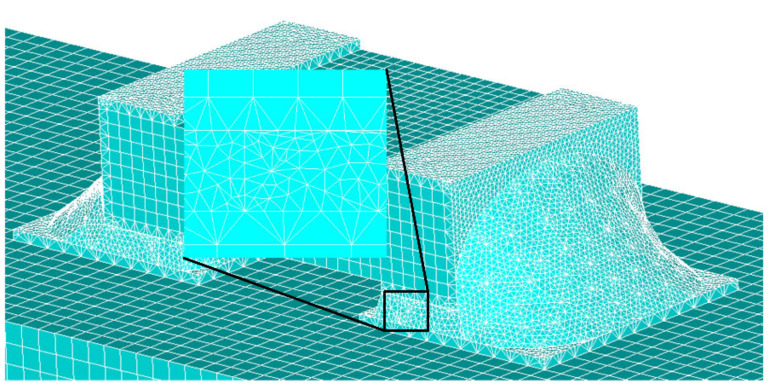
The 3-D meshed model.

For FEA mesh and boundary conditions, SOLID45 and SOLID185 hexahedron elements are selected for the ceramic capacitor body and PCB. The SOLID187 element is used for terminations and lands, and the SOLID92 element for solder joints. In the meshing process, a fine mesh pattern (Figure 6) is maintained in the solder joint as a crucial area in the analysis. In terms of boundary conditions, all degrees of freedom of the screw hole nodes at the four corners of the PCB are constrained to limit the displacement and rotation of other planes of the PCB.

**Figure 7:**
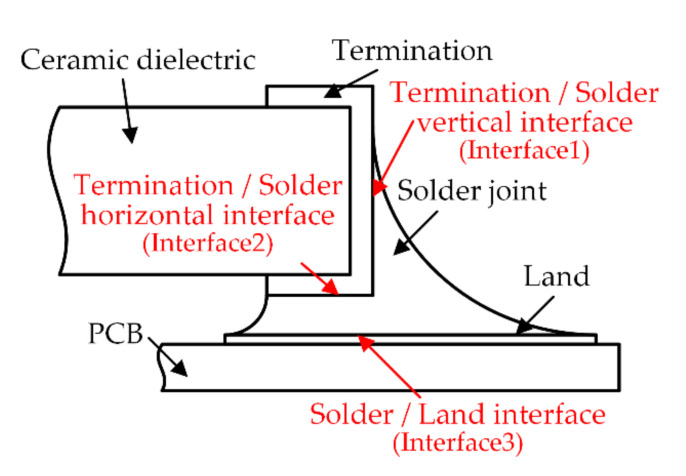
Position of a bi-material interface.

Starting from the point of contact pressure between interfaces under TC load, this paper studies the characteristics of mechanical response between interfaces. For the interface division of the surface mount solder, this paper divides it into three interfaces according to the shape of solder joints and interface contact characteristics, as shown in Figure 7; they are termination/solder vertical interface (interface 1 for short), termination/solder horizontal interface (interface 2 for short) and solder/land interface (interface 3 for short) respectively. Since both interface 1 and interface 2 are in contact with solder and capacitor termination, interface 1 and interface 2 are considered together.

The analysis model is a three-dimensional FE model. CONTA174 and TARGE170 are selected as contact elements for analysis, and contact pairs are established between solder and capacitor termination and land. Since the contact interface area of the solder joint is generally smaller than the termination and the land area, and the stiffness of the solder joint material is generally lower than that of the termination and the land material, the interface of the solder joint is set as the contact interface and the CONTA174 element is used. Termination and land are set as the target interface using the TARGE170 element. The CZM parameters for contact interfaces are shown in Table 5, where $\sigma_{\max}$ is maximum normal traction, $G_{n}^{c}$ is normal displacement jump at the completion of bonding, $\tau_{\max}$ is maximum vertical traction, $G_{t}^{c}$ is vertical displacement jump at the completion of bonding.

## 3. FEA Results and Discussion

The failure of the surface mount solder joint is accumulated by long-term damage. When the damage is added to the threshold, it will occur. The damage mainly consists of fatigue damage and creep damage. From the microscopic point of view, the grains coarsen gradually, and there are gaps between the grains, which lead to the initiation of grain boundary voids, and the continuous increase in voids leads to the initiation and expansion of cracks. From a macro point of view, thermal load causes high stress in the solder at the boundary of the contact interface, while TC load causes continuous alternating stress in these areas, leading to crack initiation and, ultimately, crack propagation until the whole interface is delaminated or the solder is broken.

**Figure 8:**
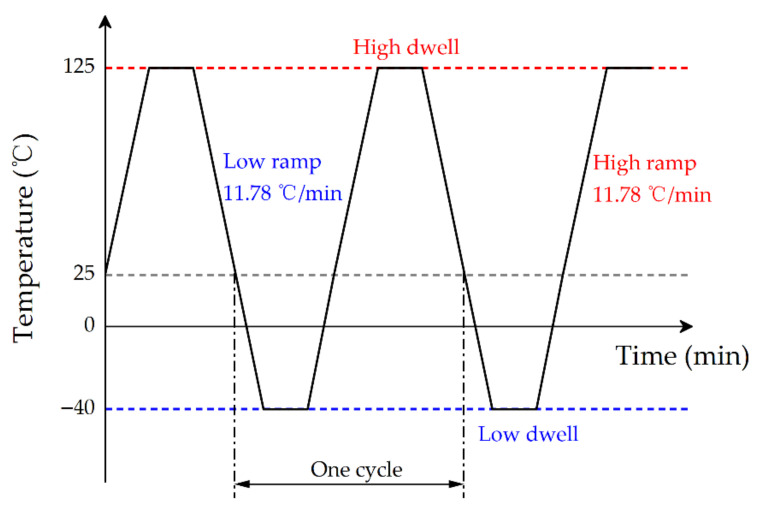
Temperature cycling profile used in the FEA.

In this paper, in order to study the mechanical response of the surface mount solder joint under TC load, the accelerated thermal cycling, as recommended by the JEDEC standard, is applied as the thermal load in the FE model [21]. The initial temperature, maximum temperature and minimum temperatures were set at 25, 125, and −45 °C, and 25 min for Low dwell and High dwell time for a 78 min per cycle and a total of 10 cycles. The temperature loading curve is shown in Figure 8. The initial geometric parameters of solder joints in FEA is the height of solder joints $h_{s}=0.66\;\mathrm{mm}$, the wetting length $l_{w}=1.1\;\mathrm{mm}$ and the gap $h_{g}=0.12\;\mathrm{mm}$.

### 3.1. Interface Delamination between SMT Solder Joint and Termination under TC Load

**Figure 9:**
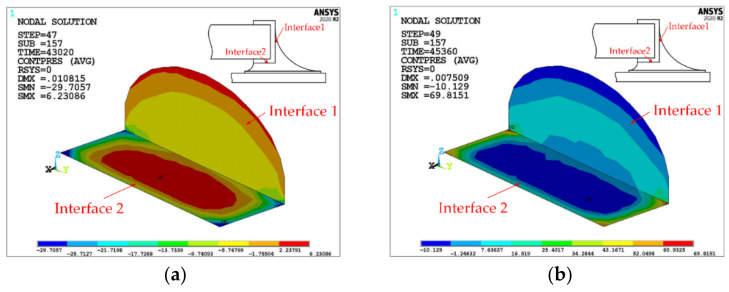
Contact pressure (unit: MPa) distribution at interface 1 and interface 2. (**a**) at 125 °C; (**b**) at −45 °C.

Contact elements are set at interfaces 1 and 2, and a TC load of 10 cycles is applied, each of which is divided into five load steps. The contact pressure contour of the capacitor termination/solder joint interface for the last cycle is shown in Figure 9. Positive values represent compression status, negative values represent tension status, and the contact pressure is normal stress.

For interface 1, under high-temperature load, the contact pressure in interface 1 changes from compression to tension with the height of the solder joint from top to bottom. Under low-temperature load, the contact pressure changes from tension to compression, with the height of the solder joint going from top to bottom. This is because th components expand, and the solder near interface 1 expands outward by heat, resulting in tension on the bottom center region of interface 1, with a delaminated trend; under low temperatures, each component shrinks, and the solder around interface 1 shrinks inwards, resulting in tension at the arc of interface 1 and a delaminated trend. Furthermore, during the cycle, the maximum tensile stress in the bottom center region of interface 1 is less than that in the top arc region of interface 1. Therefore, in interface 1, it is most likely that a delamination crack will occur in the top arc region.

For interface 2, under high-temperature load, the contact pressure changes from four corners to the center, from tension to compression. Under low-temperature load, contact pressure changes from four corners to the center and from compression to tension. This is due to the thermal expansion at high temperature and the pressure caused by the expansion of the internal solder at the four corners of interface 2, which forces the solder element to move outwards and downwards with a tendency of delamination. Under low temperatures, the component shrinks and the solder near the central region in interface 2 shrinks down to the solder, causing the interface element to be pulled here with a tendency of delamination. During the TC, the maximum tensile stress in the center area of interface 2 is less than that in the corner area of interface 2. Therefore, delamination cracks are most likely to occur in the corner area of interface 2.

**Figure 10:**
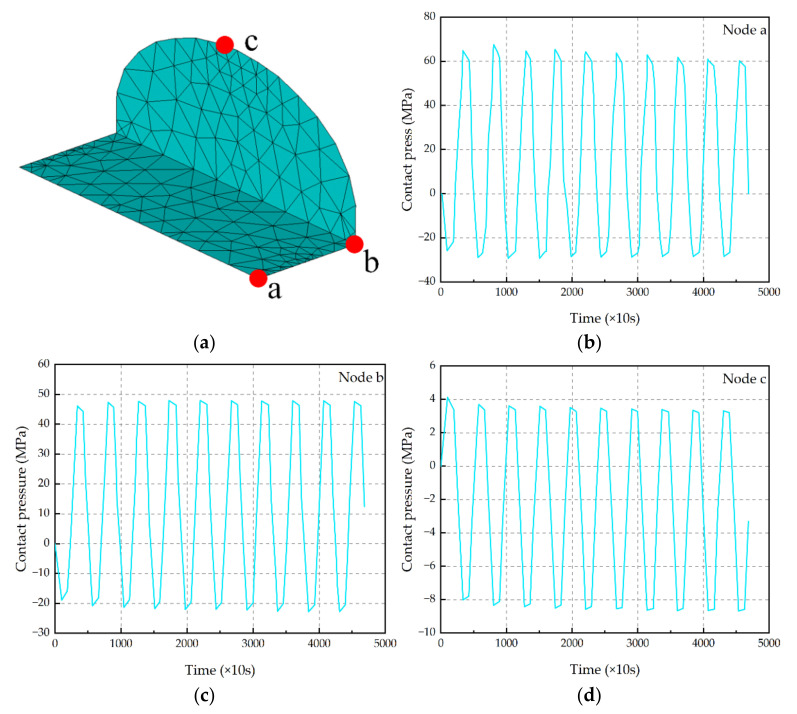
Critical points at interface 1 and interface 2 and contact pressure evolution in these critical points. (**a**) Points a, b and c are the location of the critical point; (**b**) contact pressure evolution in critical point a; (**c**) contact pressure evolution in critical point b; (**d**) contact pressure evolution in critical point c.

Three critical points for interface 1 and 2 are shown in Figure 10a. The contact pressure–time curves for the 10 temperature cycles of the three nodes are shown in Figure 10b–d. From Figure 10b–d, it can be seen that the maximum tension (i.e., the absolute value of negative contact pressure per cycle) increases with the increase in TC, which also indicates that the interface tension increases gradually at the same temperature with the increase in the number of TC, indicating that the degree of interface damage increases gradually.

### 3.2. Interface Delamination between SMT Solder Joint and Land under TC Load

**Figure 11:**
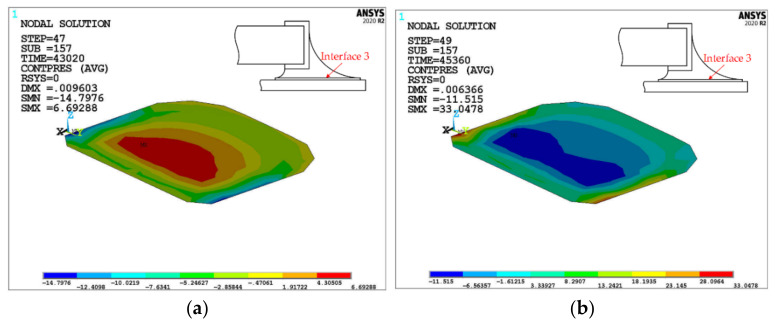
Contact pressure (unit: MPa) distribution at interface 3. (**a**) at 125 °C; (**b**) at −45 °C.

For interface 3, take the contact pressure contour of the last cycle solder joint/land interface, as shown in Figure 11. It can be seen, under high-temperature load, that the contact pressure in interface 3 changes from compression to tension from the center area to the/whole interface 3 edge. Under low-temperature load, the contact pressure changes from tension to compression from center to edge. This is because the solder near interface 3 expands outward under high-temperature load, and although the land also expands by heat, the expansion of the solder is constrained by the land because the CTE of the land is smaller than that of the solder, resulting in a tensile stress around interface 3 and a delamination trend. Under low-temperature load, the solder and land contract, and the shrinkage range of the solder is still greater than that of the land, so that the shrinkage is limited, and the solder near interface 3 shrinks inward so that the contact pressure in the central area of interface 3 is tensile stress, with a delamination trend. During a cycle, the maximum tensile stress at the y-direction side of interface 3 is slightly greater than that at the center of interface 3, and the maximum tensile stress at the center is 124.53%. Therefore, at interface 3, the overall possibility of delamination is high, but the y-direction side area is the most likely.

**Figure 12:**
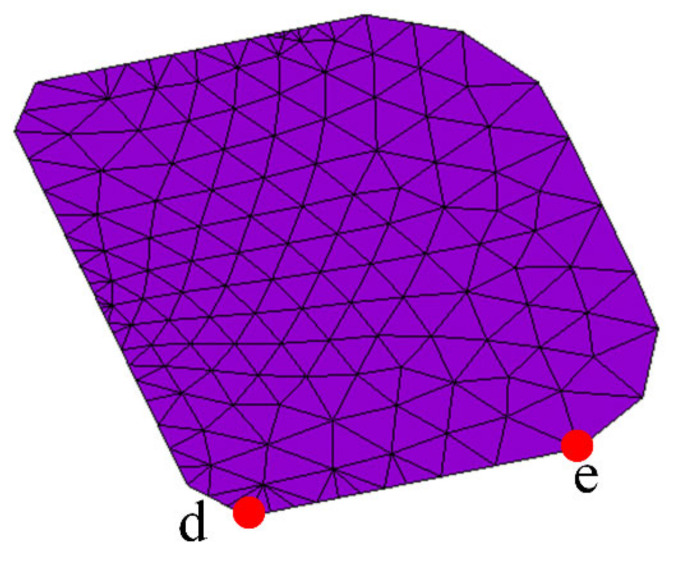
Points d and e are the location of the critical point.

**Figure 13:**
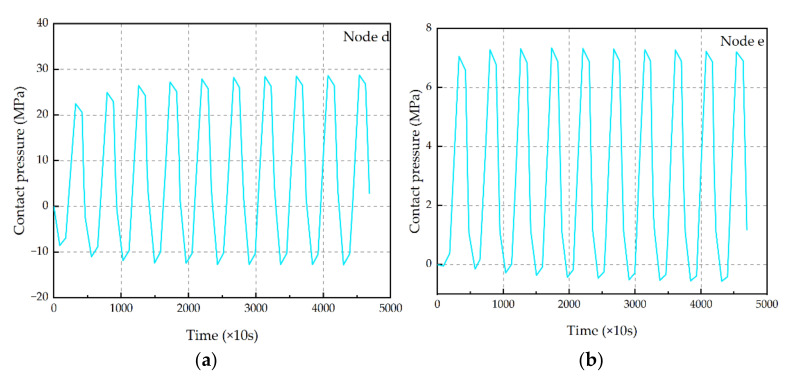
Contact pressure evolution in critical points. (**a**) Contact pressure evolution in critical point d; (**b**) contact pressure evolution in critical point e.

As shown in Figure 12, the locations of two critical nodes in Interface 3 are shown. The contact pressure–time relationship within the 10 cycles of these two critical nodes is shown in Figure 13. It can be seen from the figure that the maximum contact pressure increases with the increase in TC, which also shows that with the increase in TC times, the interface tensile stress at the same temperature is gradually increasing, indicating that the degree of interface damage is gradually increasing.

### 3.3. Discussion and Analysis

The intermetallic compound (IMC) layer is not considered in the modeling process in this paper, but because the study in this paper is based on the interface contact pressure to characterize the difficulty or trend of delamination, and the interface contact pressure is a normal stress, a proper simplification of IMC layer will not significantly change the FEA results of contact pressure. However, if the shear stress of SAC305 solder joints is studied, the influence of the IMC layer may need to be carefully considered because Andersson et al. found that the increase in IMC layer thickness is the main reason for the decrease in the shear force of the eSAC305 solder joint in the final stage of the aging period [28]. In addition, for the study of thermal aging of solder joints, the influence of the IMC layer is also critical. The thickness of the IMC layer increases linearly with the square root of the TC number, and the interface between the IMC and solder flattens gradually, while the flat IMC/solder interface decreases the fatigue life of solder joints [29].

References have used experimental approaches to study the crack propagation of solder joints [28,29,30]. The thermal aging test of the solder joint samples was carried out in the test cabinet, and the scanning electron microscope (SEM) was used to observe cracks in solder joint samples. Among them, Andersson et al.’s research has provided an important reference for this paper [28]. In their study, the crack initiation and propagation of a reflow surface mount solder joint under TC load were tested experimentally. They observed that for reflow soldered 0805 components, the majority of all cracks either propagate vertically along the metal termination of the component or horizontally under the component, and these locations are consistent with the interfaces analyzed in this paper. At the same time, their observations show that the crack generally occurs at the termination/solder interface after 5000 TCs. When more cycles occur, a crack occurs at the solder/land interface until the solder joint completely fails, that is, a crack propagated from one side of the solder joint to the other. The contact pressure analysis in this paper also explains Andersson et al.’s observation that the tensile stress at the termination/solder interface is almost greater than that at the solder/land interface [28]. It should be noted that this test method can only be used to observe the initiation and propagation of cracks that have occurred after several TC loads and cannot explore the mechanical behavior of the interface, which is compensated by the contact pressure study in this paper.

## 4. Adaptive Surrogate Model-Based SMT Geometry Parameter Optimization

The shape of the solder joint has a significant effect on the interface contact pressure, and a small size fluctuation will change the maximum contact pressure value at the interface of the solder joints, which will affect the interface crack initiation earlier or the expansion of the solder joint to generate a larger crack, which will ultimately lead to the failure of the solder joint. The objective of this section is not to limit the contact pressure at the critical points but to shift to the average contact pressure values in the zones prone to interface delamination (as analyzed in Section 2). This not only reduces the contact pressure of the interface critical points, making it not easy to delaminate but also reduces the contact pressure of the nearby area, which can reduce the risk of further crack propagation even if cracks occur in the solder joint.

### 4.1. A Brief Review: Kriging Surrogate Model and EGO Method

Th Kriging surrogate model (which is more commonly called the Gaussian process in the emerging field of artificial intelligence) is a high-precision interpolated response surface model that uses the sum of regression functions and random processes to represent the functional relationship between design variables and response variables [31]. The biggest difference between the Kriging surrogate and the traditional polynomial response surface model is that it uses statistical assumptions to interpret the results of the surrogate model. The expression of the Kriging model is:

(8) $$\begin{array}{cc} Y(x) & =\sum_{m=1}^{n}\beta_{m}f_{m}(x)+s\left(x\right)\\ & =\hat{y}(x)+s\left(x\right) \end{array}$$

where, $f_{m}(x)$ is the basic function of the Kriging surrogate, and the polynomial basis function is generally used. $\beta_{m}$ is the regression coefficient; $\sum_{m=1}^{n}\beta_{m}f_{m}(x)$ represents the expectation of random function Y(x); $s\left(x\right)$ is a static random process with a mean of 0 and a variance of $\sigma_{z}^{2}$. Physically speaking, the first term $\sum_{m=1}^{n}\beta_{m}f_{m}(x)$ roughly represents the overall trend of the model, giving the general position of the real model at the prediction point. The second term $s\left(x\right)$ represents the spatial correlation of the relevant parameters in the design space, giving the local deviation of the model at the prediction point.

It is worth emphasizing that, compared with other surrogate models, such as polynomial response surface, Kriging can not only provide the prediction response but also provide the uncertainty of the prediction. This property can be very useful for adaptive optimization. Jones et al. proposed the Efficient Global Optimization (EGO) algorithm, which uses a so-called adaptive learning function (infill criterion) to achieve sequential optimization, i.e., the Kriging surrogate model can provide the predictive uncertainty measures, guide the newly generated sample points, update the surrogate model. Therefore, the EGO algorithm can greatly improve the efficiency of surrogate optimization. The core of the EGO algorithm is to add the training samples of the initial Kriging surrogate model according to the specific infill criterion and gradually improve the accuracy of the Kriging surrogate model near the optimal solution. The infill criterion plays a decisive role in the success or failure of the optimization and the efficiency of the optimization. Maximizing the expected improvement function, also known as the EI Criterion, is used in the EGO algorithm. Jones’ paper can be used for other details [32].

According to the EI criteria, for a certain prediction point *x*, the amount of improvement provided by the EI criterion can be expressed as:

(9) $$I\left(x\right)=\left\{\begin{array}{ll} f_{\min}-Y\left(x\right) & \mathrm{if}Y\left(x\right)<f_{\min}\\ 0 & \mathrm{otherwise} \end{array}\right.$$

where $y_{\min}$ is the optimal value of the true objective function for all sample points at the current iteration. Correspondingly, the EI function is defined as

(10) $$EI(x)=\int_{-\infty }^{f_{\min}}\left(f_{\min}-Y\right)\frac{1}{\sqrt{2\pi }s(x)}\exp(-\frac{\hat{y}{\left(x\right)}^{2}}{2s{\left(x\right)}^{2}})dY$$

Solving this integral gives the expression of the EI function as:

(11) $$EI(x)=(f_{\min}-\hat{y}(x))\Phi (\frac{f_{\min}-\hat{y}(x)}{s(x)})+s(x)\phi (\frac{f_{\min}-\hat{y}(x)}{s(x)})$$

where, ϕ(⋅) and Φ(⋅) represent the probability density function (PDF) and the cumulative distribution function (CDF) of the standard normal distribution. It can be seen that the EI function takes into account both the mean $\hat{y}(x)$ of the predicted value of the Kriging model and the influence of the prediction point error s(x). Therefore, the new sample points determined by the EI criterion can be used for both a rough search of the whole design space and a fine search of local optimal points, which means the EGO algorithm can be more suitable for complex problem optimization.

### 4.2. IEGO: An Improved Adaptive Optimization Method

**Figure 14:**
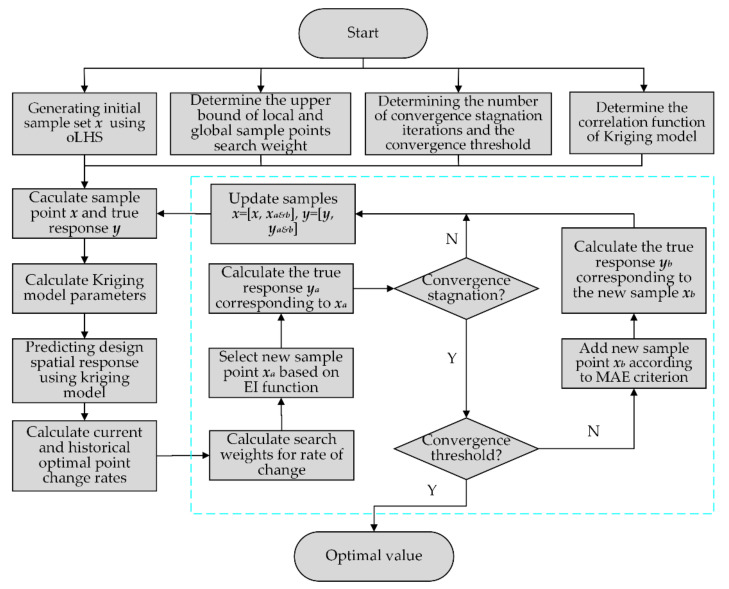
The IEGO algorithm flowchart.

Equation (11) shows that the weight assigned by the traditional EGO algorithm to the first and second terms of the EI function is one, which means that during the whole optimization search process, when the current optimal point continues to converge, the EGO algorithm cannot determine whether the current optimal point is a global optimal point, so it may fall into the trap of local convergence or lead to very slow convergence later. Therefore, this paper presents an improved EGO (IEGO) algorithm: (1) at the beginning of the search to enhance the search of global sample points and expand the search scope; (2) in the search process, Kriging’s current optimal change range is associated with the search weights of local and global sample points, that is, when the change range is large, the global search weight will also increase, expanding the global search ability. When the optimal change range is small, it means that the IEGO algorithm may find the global optimum and need to refine the local search, so the global search weight will also decrease synchronously; (3) When the convergence stops, increase the sample points (marked as Minimize Absolute Error, MAE criterion) with a large error between the predicted and true values of the surrogate model to improve the accuracy of the Kriging surrogate model. The pseudo-code for IEGO is as follows, and the flow is shown in Figure 14.

**Step 1**: Determining the upper search weight limits for local and global sample points, determining the stagnation convergence algebra and convergence threshold, and the correlation function $R(x_{i},x_{j})$ of the Kriging surrogate model;

**Step 2**: Determine the initial sample points ***x*** (using Optimal Latin Hypercube Sampling) and the corresponding outputs ***y***;

**Step 3**: Using the initial sample to generate the Kriging surrogate model, the parameters of the Kriging model, i.e., the weighting factor $\beta_{j}$, are calculated. The DACE (Design and Analysis of Computer Experiments) toolbox is used to generate the Kriging surrogate model in this paper;

**Step 4**: Based on the EI criterion and the numerical optimization algorithm (e.g., DE), the Kriging surrogate model is used to determine the EI function in the design space;

**Step 5**: Calculate the current and historical average optimal change rates, and determine the corresponding search weight. The corresponding relationship between the two can be set as linear; that is, when the change rate is greater than the set stagnation threshold, the search weight is reset to the maximum. When the change rate decreases, the search weight decreases synchronously. When the change rate is close to 0, the search weight is reset to the minimum value of 0; that is, the RMSE infill criterion is adopted, the optimum model of the RMSE infill criterion is:

(12) $$Maximize:RMSE\left(x\right)=s\left(x\right)$$

**Step 6**: Determine the new sample point ***x_a_*** and calculate the corresponding true output ***y_a_***;

**Step 7**: Determine if the result iteration is stalled. If convergence occurs, skip step (8), otherwise skip **Step 10**;

**Step 8**: Determine if the convergence threshold is met. If satisfied, skip step (11), otherwise skip **Step 9**;

**Step 9**: Add a new sample point ***x_b_*** according to the MAE criterion and calculate the corresponding real output ***y_b_***;

**Step 10**: Update the sample set ***x*** = [***x***, ***x_a_***_&***b***_], ***y*** = [***y***, ***y_a_***_&***b***_], and then skip **Step 3**;

**Step 11**: Output the optimal solution and the true value.

**Figure 15:**
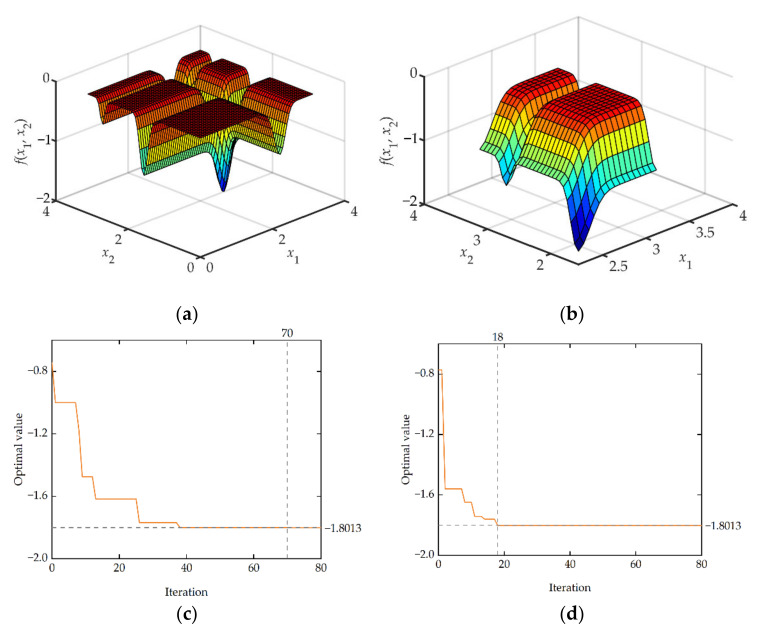
Benchmark function image and optimization convergence profile: (**a**) 2-D Michalewicz function; (**b**) local schematic of Michalewicz function; (**c**) convergence profile of traditional EGO algorithm; (**d**) convergence profile of IEGO algorithm.

**Figure 16:**
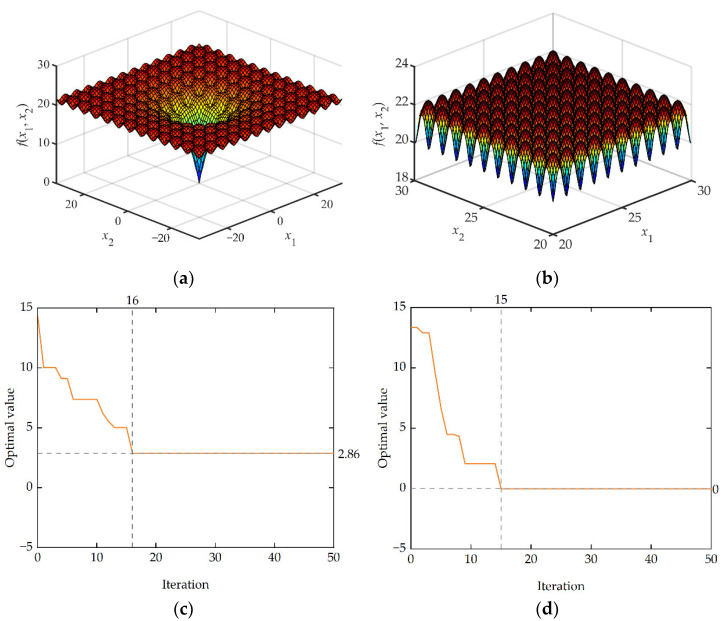
Benchmark function image and optimization convergence profile. (**a**) The 2-D Ackley function; (**b**) local schematic of Ackley function; (**c**) convergence profile of traditional EGO algorithm; (**d**) convergence profile of IEGO algorithm.

This paper uses two benchmark functions, Michalewicz (Figure 15a,b) and Ackley (Figure 16a,b), to test and verify the efficiency and accuracy of IEGO. The specific expressions for benchmark functions, test characteristics, and algorithm settings for this article are given in Appendix B.

For the Michalewicz function, both the traditional EGO algorithm and the IEGO algorithm have found the global optimal. However, since the Michalewicz function is multimodal and has several local minima, the iteration curve of the algorithm shows that the traditional EGO algorithm has been trapped in the local minima several times and hovered in it, resulting in a very slow convergence rate until the 70th iteration reaches the global optimal (Figure 15c). In contrast, because the IEGO algorithm proposed in this paper has strong resistance to the local optimum, its convergence rate is significantly faster than that of traditional EGO and finally converges to a global optimum (Figure 15d).

As mentioned in Appendix B, the Ackley function has special properties (a deep hole in the center of a nearly flat outer region, with a large number of local minima distributed at the same time). The Ackley function value under the traditional EGO algorithm can approach the global optimum at the early stage of the optimization phase (Figure 16c), which is almost impossible for the hill-climbing algorithm to complete, but it will converge locally after several iterations and cannot jump out of local minima. After falling into local convergence, the Ackley function value under the IEGO algorithm can further improve the accuracy of the Kriging surrogate model by adjusting the global search weight and using the RMSE infill criterion, jump out of local convergence quickly, and finally, reach the global optimal (Figure 16d).

From the above numerical examples, it can be seen that the IEGO method proposed in this paper has the advantages of fast optimization speed and strong global optimization ability compared with the traditional EGO algorithm.

### 4.3. Optimization of Interface Contact Pressure

**Figure 17:**
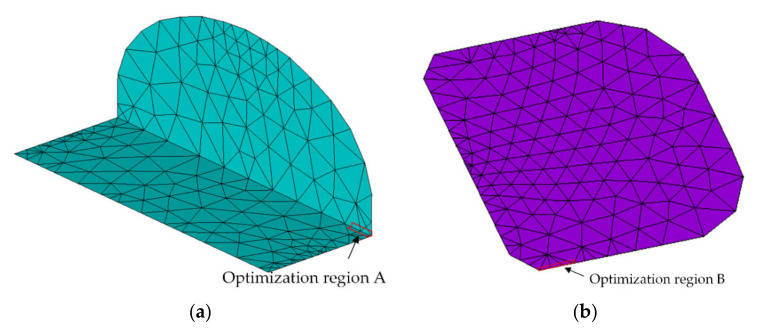
Contact pressure optimization region. (**a**) Optimized region of interfaces 1 and 2 (i.e., the interface between termination and solder joint); (**b**) optimized region of interface 3 (i.e., the interface between land and solder joint).

SMT solder joints are easy to be delaminated between pins and lands of components under the thermal load in a service environment. The shape of solder joints has a significant and complex effect on the interface delamination. Therefore, the optimum variables in this paper are the height of solder joints $h_{s}$, the wetting length $l_{w}$ and the gap $h_{g}$. The interface contact pressure can directly reflect the difficulty of interface delamination, so the interface contact pressure is the optimization target. At the same time, this paper optimizes not only for dangerous points but also for dangerous areas. According to the analysis in Section 2, for interface 1 and interface 2, it is easier for solder joints to be delaminated at the interface between the two interfaces and the terminations. In this paper, the maximum contact pressure at the solder of interface 1 and interface 2 and the average contact pressure of all nodes with a width of 0.02 mm and a depth of less than 0.06 mm are predicted. The optimization range is shown in Figure 17a. At the same time, for interface 3, solder joints are most easily stratified on both sides of interface 3 and the land. The maximum contact pressure on both sides of interface 3 and the average contact pressure of all nodes with a width of less than 0.1 mm are also optimized in this paper. The optimization region is shown in Figure 17b.

Therefore, the target of optimization is not only the contact pressure $p_{b}$ and $p_{d}$ of nodes b and d, but also the average contact pressure $p_{A}$ and $p_{B}$ of all nodes in delaminated area A and B. Because the four indexes have different degrees of influence on the SMT solder delamination, the weighting coefficients $a_{1}$, $a_{2}$, $a_{3}$ and $a_{4}$ are introduced, and the sum of the weighting coefficients is 1, based on the analysis of contact pressure of dangerous nodes in Section 2, the weighting coefficients of each item are set to $a_{1}=0.4$, $a_{2}=0.2$, $a_{3}=0.3$, $a_{4}=0.1$, respectively. Establish optimization model:

(13) $$\begin{array}{l} find\\ \;h_{s},l_{w},h_{g}\\ to\\ \;Maximum\;f=a_{1}p_{b}+a_{2}p_{d}+a_{3}p_{A}+a_{4}p_{B}\\ s.t.\\ \;h_{s\min}\leq h_{s}\leq h_{s\max}\\ \;l_{w\min}\leq l_{w}\leq l_{w\max}\\ \;h_{g\min}\leq h_{g}\leq h_{g\max}\\ \;a_{1}+a_{2}+a_{3}+a_{4}=1 \end{array}$$

where $h_{s\min}=0.62\;\mathrm{mm}$ and $h_{s\max}=0.72\;\mathrm{mm}$ represent the extreme value of the interval of the solder height, respectively. $l_{w\min}=0.9\;\mathrm{mm}$ and $l_{w\max}=1.4\;\mathrm{mm}$ represent the extreme values of the wetting length of the solder joints, respectively. $h_{g\min}=0.09\;\mathrm{mm}$ and $h_{g\max}=0.14\;\mathrm{mm}$ represent the extreme values of the interval between the gap of the solder joints, respectively.

**Figure 18:**
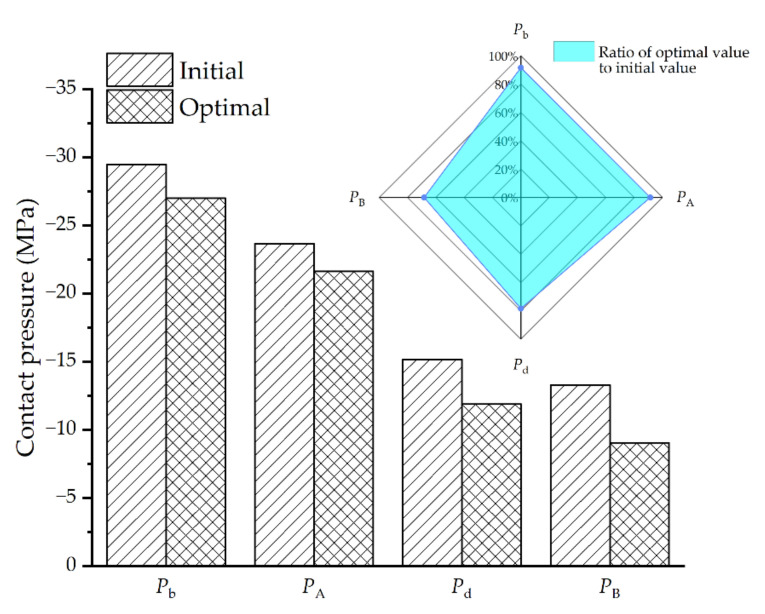
Optimization results and comparison.

Based on the IEGO algorithm proposed in this paper, the above optimization model is solved, and the contact pressure values of each node and critical region in the optimization model are obtained by the high-cost FEA described above. The optimum geometric parameters of the solder joint are shown in Table 6. Correspondingly, the contact pressure of each critical point and critical region under this optimal combination of parameters is shown in Figure 18. Specifically, the contact pressure of $p_{b}$ is −27.01 MPa, $p_{A}$ is −21.63 MPa, $p_{d}$ is −11.89 MPa, $p_{B}$ is −9.06 MPa, compared with the initial geometric parameters, the contact pressure of $p_{b}$ decreases by 8.38%, that of $p_{A}$ by 8.62%, that of $p_{d}$ by 21.6%, and that of $p_{B}$ by 31.7%.

## 5. Conclusions

The FE model for SMT packaging has been established, in which the geometry of the solder joint was generated based on the minimum energy method, so the geometry and energy constraints in the actual process were considered to make it more conform to the actual shape after reflow. Secondly, the distribution of contact pressure at the solder interface under TC load is analyzed. Due to the different CTE between materials, the maximum contact pressure occurs in the top arc region at interface 1, the corner region at interface 2, and the y-direction side region at interface 3, which means that these regions are prone to interface delamination under TC load. After that, an improved algorithm (IEGO) is presented, which is an adaptive optimization method based on the surrogate model. The benchmark functions prove the superiority of the improved algorithm. The improved algorithm is used to optimize the contact pressure of critical regions (no longer just critical points) between solder interfaces. The optimal combination of parameters after optimization is $h_{s}=0.703\;\mathrm{mm}$, $l_{w}=1.288\;\mathrm{mm}$, $h_{g}=0.125\;\mathrm{mm}$. At this time, the contact pressure of critical points $p_{b}$ and $p_{d}$ and critical regions $p_{A}$ and $p_{B}$ decreases by 8.38%, 21.6%, 8.62%, 31.7%, respectively. The results show that the contact pressure of the SMT solder joint decreases significantly; that is, the possibility of delamination decreases.

## Figures and Tables

**Table 5:** CZM parameters for SAC305 [27].

Parameter	$\sigma_{\max}\;(\mathrm{MPa})$	$G_{n}^{c}{\;(J/m}^{2})$	$\tau_{\max}\;(\mathrm{MPa})$	$G_{t}^{c}{\;(J/m}^{2})$
value	47.5	480	56	600

**Table 6:** Optimum geometric parameter of the solder joint.

Geometric Parameter	$h_{s}\left(\mathrm{mm}\right)$	$l_{w}\left(\mathrm{mm}\right)$	$h_{g}\left(\mathrm{mm}\right)$
Optimal value	0.703	1.288	0.125

**Figure A1:**
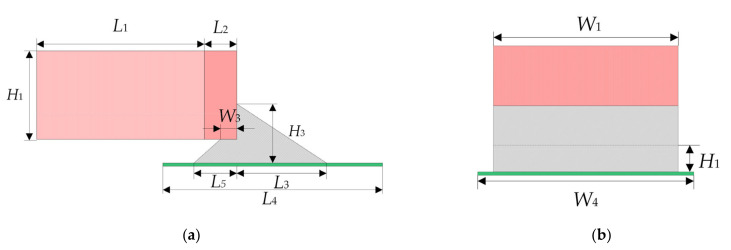
Initial shape of solder joint. (**a**) main view; (**b**) side view.

**Figure A2:**
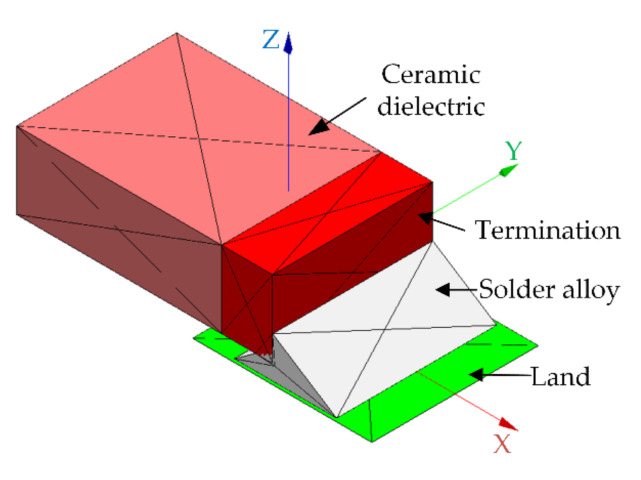
The initial shape of the solder is established in the surface evolver.

## Data Availability

Not applicable.

## Appendix A

### Minimum Energy-Based Solder Joint Geometry Modeling

According to the principle of minimum energy, the total energy E of the solder joint system is equal to the sum of surface potential energy $E_{s}$, external force potential energy $E_{f}$ and the potential energy of the solder joint $E_{g}$ (such as gravity, etc.). The energy control equation is as follows:

(A1) $$E=E_{s}+E_{g}+E_{f}$$

In this formula, the surface potential energy of the interface can be expressed as:

(A2) $$E_{s}=\iint_{A_{0}}TdA+\sum_{i=1}^{N}\iint_{A_{1}}T_{i}dA$$

where $A_{0}$ is the surface of solder exposed to air, T is the surface tension of liquid solder (melted) in contact with air, N is the number of solid surface interfaces between the solder and device, $A_{1}$ is the contact area between solid and liquid, $T_{i}$ is the contact tension at the solid–liquid interface.

The external force potential energy $E_{f}$ can be expressed as:

(A3) $$E_{f}=\sum_{j=1}^{M}F_{j}\cdot U_{j}$$

where M is the total number of external forces, $F_{j}$ is the external force to the system, and $U_{j}$ is the displacement under the external force $F_{j}$.

When $E_{g}$ is only gravitational potential energy, then:

(A4) $$E_{g}=\underset{V}{∭}\rho gzdV$$

where ρ is the density of the solder alloy, g is the acceleration of gravity, z is the height coordinate, V is the volume of the solder alloy.

Therefore, according to the energy control equation, when the solder joint system is subjected to external forces, the overall energy of the system E is:

(A5) $$E=E_{s}+E_{f}+E_{g}=\iint_{A_{0}}TdA+\sum_{i=1}^{N}\iint_{A_{i}}T_{i}dA+\sum_{j=1}^{M}F_{j}\cdot U_{j}+\underset{V}{∭}\rho gzdV$$

The energy control equation is determined. In order to obtain the lowest energy solder joint system, constraints are required. For a solder joint, the geometric constraints are: (a) When the solder joint changes from melting state to solidification state, the total volume of the whole process remains unchanged. (b) Solder cannot be dissolved into other solids. Thus, by solving the optimal solution problem of minimizing the overall energy of the system, the geometric shape of the solder joint can be solved.

Specific to the object of this study, the basic assumptions are as follows:

1. There is no penetration between the land and the solder.
2. There are no holes or other defects inside the solder materials.
3. During the wetting process, the properties of the solder materials are stable, and there is no change in properties.
4. The total solder volume is not affected by external factors and remains unchanged.

The initial model built in the surface evolver is shown in Figure A1, and the geometric parameters are shown in Table A1.

**Table A1:** Initial parameters set in the surface evolver.

Component	Parameter	Variable	Initial Value (mm)
Ceramic dielectric	Length	$L_{1}$	2
	Width	$W_{1}$	1.25
	Thickness	$H_{1}$	0.6
Metal termination	Length	$L_{2}$	0.4
Solder	Length	$H_{3}$	0.32
	Extension length	$L_{3}$	0.6
	Internal length	$L_{5}$	0.2
	Width	$W_{3}$	0.13
Land	Length	$L_{4}$	1.4
	Width	$W_{4}$	1.3
	Gap between land and termination	$H_{2}$	1.2

Based on the above parameters, the initial shape of the solder is established in the surface evolver, as shown in Figure A2. The light red and dark red parts in the model represent surface mount components such as capacitors or resistors. The light red part represents ceramic passivation, and the dark red part represents metal termination on both sides of the surface mount component. The solder alloy can be wet on termination (dark red parts) and not on other surfaces (light red parts) of the component. The green rectangle below represents the land on the PCB on which the solder alloy can also be wetted. The properties of solder alloy itself, such as surface tension (liquid), density and wetting angle, are also important parameters that affect the final shaping state of the solder joint. The SAC305 lead-free solder alloy is selected in this paper. When the solder alloy melts into a liquid state, the surface tension is 560 mN/m, the density is 7.8 g/cm^3^, the wetting angle with metal termination is 30 degrees, and the wetting angle with copper land is 36 degrees [33,34].

## Appendix B

### Benchmark Functions

1. Michalewicz function

The Michalewicz function has d! local minima, which is multimodal. Parameter *m* defines the steepness of valleys and ridges; the larger the *m*, the more difficult it is to search. The recommended value of *m* is 10. The expression is:

(A6) $$f(x)=-\sum_{i=1}^{d}\sin(x_{i})\sin^{2m}\left(\frac{ix_{i}^{2}}{\pi }\right)$$

where *d* defines the function dimension. This paper uses the 2-D form, $x_{i}\in \left[0,\;\pi \right]$. The global minimum value is −1.8013, and the corresponding minimum value is $\left(2.20,1.57\right)$. In this paper, the correlation function of the Kriging surrogate model is a Gaussian correlation function, the stagnation convergence algebra is set to 3, and the convergence threshold is 0.0001. In addition, the number of initial sampling points is 21, and the sampling method is optimum Latin Hypercube Sampling (oLHS). In the traditional EGO algorithm, the single point EI criterion is used to add update points, which are determined by the differential evolution (DE) algorithm.

2. Ackley function

The Ackley function is one of the commonly used functions to test the performance of optimization algorithms. Its characteristic is a deep hole in the center of a nearly flat outer region, with a large number of local minima distributed at the same time. This function is particularly easy to induce optimization algorithms, especially hill-climbing algorithms, to fall into one of its many local minima. The expression is:

(A7) $$f(x)=-a\exp\left(-b\sqrt{\frac{1}{d}\sum_{i=1}^{d}x_{i}^{2}}\right)-\exp\left(\frac{1}{d}\sum_{i=1}^{d}\cos\left(cx_{i}\right)\right)+a+\exp\left(1\right)$$

where *a* = 20, *b* = 0.2, *c* = 2π, $x_{i}\in \left[-32.768,\;32.768\right]$. The global minimum value is 0, and the corresponding minimum value point is $\left(0,\cdots ,0\right)$. In this paper, the 2-D expression of the Ackley function is taken, which is generally marked as Ackley2. Other parameters are the same as those in the example of the Michalewicz function.
